# Non-Interventional Weight Changes Are Associated with Alterations in Serum Uric Acid Levels

**DOI:** 10.3390/jcm13082314

**Published:** 2024-04-17

**Authors:** Shiri Weinstein, Elad Maor, Jonathan Bleier, Alon Kaplan, Tammy Hod, Avshalom Leibowitz, Ehud Grossman, Gadi Shlomai

**Affiliations:** 1 Internal Medicine D and the Hypertension Unit, Sheba Medical Center, Tel-Hashomer, Ramat Gan 5262504, Israel; shiri.weinstein@sheba.health.gov.il (S.W.); yeonatan.bleier@sheba.health.gov.il (J.B.); alon.kaplan@sheba.health.gov.il (A.K.); avshalom.leibowitz@sheba.health.gov.il (A.L.); 2 Tel Aviv Faculty of Medicine, Tel-Aviv University, Tel-Aviv 6997801, Israeltamar.hod@sheba.health.gov.il (T.H.); 3 Leviev Heart Center, Sheba Medical Center, Tel-Hashomer, Ramat Gan 5266202, Israel; 4 The Institute of Endocrinology, Diabetes and Metabolism, Sheba Medical Center, Tel-Hashomer, Ramat Gan 5262504, Israel; 5 Renal Transplant Center, Sheba Medical Center, Tel-Hashomer, Ramat Gan 5262504, Israel; 6 Nephrology Department, Sheba Medical Center, Tel-Hashomer, Ramat Gan 5262504, Israel; 7 Adelson School of Medicine, Ariel University, Ariel 4070000, Israel; ehud.grossman@ariel.ac.il

**Keywords:** uric acid, weight changes, body mass index, BMI

## Abstract

**Background/Objectives**: Serum uric acid is an established cardiovascular risk factor. Higher serum uric acid levels are associated with overweight and obesity. We assessed whether non-interventional weight changes affect serum uric acid levels. **Methods**: We performed a retrospective analysis of 19,193 participants referred to annual medical screening. Body mass index (BMI) and serum uric acid were measured annually. Subjects were divided into five groups according to changes in BMI between visits: large reduction (reduction of more than 5% in BMI), moderate reduction (reduction of more than 2.5% and 5% or less in BMI), unchanged (up to 2.5% change in BMI), moderate increase (increase of more than 2.5% and 5% or less in BMI), and large increase (increase of more than 5% in BMI). The primary outcome was serum uric acid level changes between visits. **Results**: A decrease in serum uric acid levels was evident as BMI decreased and an increase in serum uric acid levels was associated with an increase in BMI. The proportion of patients whose serum uric acid levels were increased by at least 10% between visits increased with the relative increase in BMI, while the proportion of patients whose serum uric acid levels were reduced by at least 10% decreased with the relative decrease in BMI. **Conclusions**: Non-interventional weight changes, even modest, are associated with significant alterations in serum uric acid levels. Our findings may aid in better risk stratification and the primary prevention of cardiovascular morbidity and mortality.

## 1. Introduction

Obesity and overweight are well-established cardiovascular risk factors and are associated with an increased prevalence of dyslipidemia, diabetes mellitus, hypertension, and atherosclerotic cardiovascular-related morbidity and mortality [1,2,3,4]. Weight gain is associated with an increased prevalence of cardiometabolic risk factors such as high triglycerides and reduced high-density lipoprotein cholesterol [5], as well as impaired glucose homeostasis [6,7,8]. Achieving and maintaining a lower body weight reduces cardiovascular risk among overweight and obese patients [9], and the benefits achieved are often preserved, even following weight regain [10].

Overweight and obesity predispose individuals to increased uric acid production and a decrease in uric acid renal clearance and are thereby associated with hyperuricemia [11,12]. Serum uric acid is considered an emerging cardiovascular risk factor [13,14]. Hyperuricemia causes oxidative stress due to nitric oxide production inhibition; endothelial dysfunction due to the deposition of uric acid in vascular walls, which induces smooth muscle proliferation; and renin-angiotensin system activation [15]. In addition, hyperuricemia causes the increased oxidation of LDL, thus contributing to the development of atherosclerosis and its complications [16]. It also increases arterial inflammation and arterial stiffness, which contribute to hypertension development, and induced endoplasmic reticulum stress [13]. 

Elevated serum uric acid levels correlate with higher body weight, disrupted glucose homeostasis, hypertension, dyslipidemia, atherosclerosis, chronic kidney disease, and metabolic syndrome [17,18,19,20,21]. Serum uric acid variability is associated with the development of ischemic heart disease and all-cause mortality [22,23,24]. Even without overt hyperuricemia, higher serum uric acid levels are associated with cardiovascular-related mortality, as well as all-cause mortality [25], and lowering serum uric acid into the normal range correlates with a reduction in all-cause mortality and cardiovascular-related death [26,27]. Moreover, a recent study shows that the addition of serum uric acid to cardiovascular risk score models significantly improves their accuracy [28]. 

Among obese and overweight patients, weight loss correlates with reductions in serum uric acid [29]. However, most studies, to date, focus on the association of various interventional weight loss programs on serum uric acid [30,31,32], while there is paucity of data regarding the effects of minor, non-interventional weight changes on serum uric acid. Most clinic patients do not participate in a structured weight loss program, yet their weight may change, intentionally or not. We, therefore, sought to explore the effects of non-interventional weight changes on serum uric acid regardless of any dietary restriction or nutritional habits. 

## 2. Materials and Methods

**Study population**: Our study population was enrolled from the registry of the Medical Screening Institute at Chaim Sheba Medical Center, between the years 2000 and 2020.

As previously described [33,34], every clinic visit included a completion of a standard questionnaire regarding participants’ medical history, any recent medical events since their previous visit, and their demographic characteristics, as well as lifestyle and health-related habits. The weight and height of all subjects, wearing light clothes without shoes, were measured and recorded at each visit. BMI was calculated as weight in kilograms divided by the squared height in meters. Weight changes between visits, if they occurred, were all subject-driven and no weight loss intervention program was applied. Following an eight hour fast, venous blood for analysis, including serum uric acid levels, was drawn by a trained nurse. 

The Chaim Sheba Medical Center Institutional Helsinki Committee approved this study. As only retrospective data were recorded anonymously, the need for informed consent was waived by the committee. 

**Inclusion and exclusion criteria**: The whole database included 176,621 clinic visits. We included every patient who had two consecutive annual clinic visits. If there were more than two consecutive annual clinic visits, we analyzed only the first two encounters. Overall, 21,111 individuals met the inclusion criteria. Subjects were excluded from analysis if their height, weight, or serum uric acid levels were missing, or if they had extreme BMI values (less than 15 kg/m^2^ or more than 50 kg/m^2^) or extreme serum uric acid levels (less than 1 mg/dL or more than 15 mg/dL). After patient exclusion, the final study cohort comprised 19,193 subjects.

**Definitions and outcome**: Participants were divided according to the percent change in BMI between the first and second visits: BMI reduction of more than 5% (“large reduction”), BMI reduction between 2.5% and 5% (“moderate reduction”), BMI reduction of less than 2.5% or elevation of less than 2.5% (“unchanged”), BMI elevation between 2.5% and 5% (“moderate increase”), and BMI elevation of more than 5% (“large increase”). The primary outcome was the change in serum uric acid levels between the first and second visit. 

**Statistical analysis**: Trends in characteristics for categorical variables were assessed using a chi-squared test. A logistic regression model was calculated to assess the relationship between baseline characteristics and increases of at least 10% in serum uric acid levels in the second visit. BMI change; gender; and diagnosis of ischemic heart disease (IHD), hypertension (HTN(, or diabetes mellitus (DM) were tested individually and in a multivariable logistic regression model as clinically and epidemiologically relevant variables. Subset analysis was performed for gender and baseline BMI. All analyses were performed using R software (R Development Core Team, Vienna, Austria, version 4.1.0) [35]. A two-sided *p*-value < 0.05 was used for statistical significance.

## 3. Results

The final analysis included 19,193 patients. The baseline demographic and clinical characteristics according to the pre- specified BMI change groups are presented in Table 1. Patients in the “large increase” group were younger and more likely to be females compared to other BMI change groups (Table 1). The prevalence of hypertension, ischemic heart disease, and diabetes mellitus was similar between the pre-specified groups of BMI change (Table 1).

Table 2 describes the BMI and serum uric acid levels for visit 1 and visit 2 across all pre-specified groups. The mean baseline BMI was 26 kg/m^2^. No statistically significant changes in BMI were noted between visits across the entire study cohort. However, 23.3% had increased their BMI by more than 2.5% and 20.8% had reduced their BMI by no less than 2.5%. In addition, 9.2% of patients had increased their BMI by 5% or more, whereas 9.3% of patients had reduced their BMI by at least 5% (Table 2).

**Figure 1:**
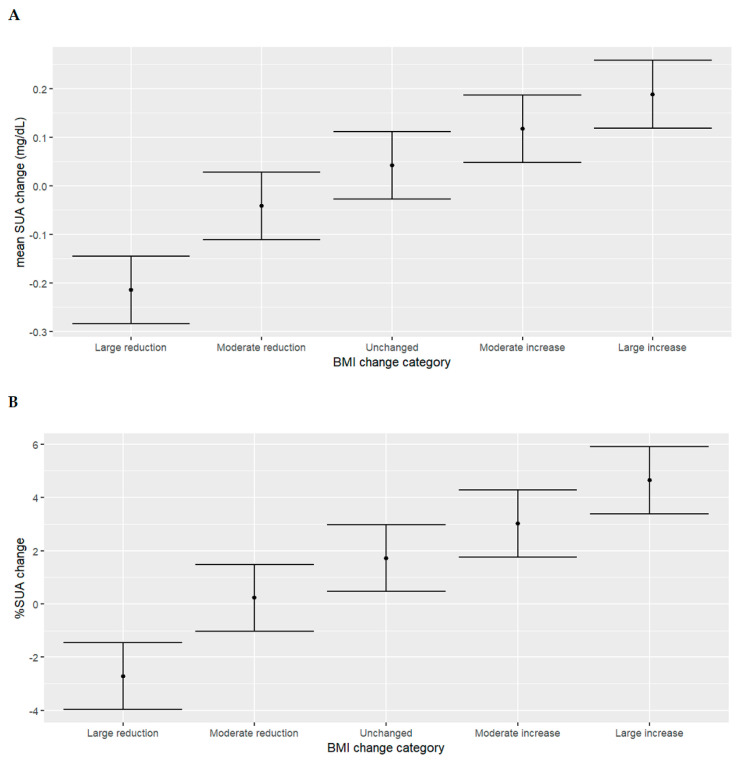
Mean serum uric acid change ((**A**)—mg/dL, (**B**)—percentage) according to the pre-specified BMI change groups. The black dots represent mean serum uric acid change (mg/dL or percentage); bars represent standard deviation. Abbreviations: BMI, body mass index; SUA, serum uric acid.

For the entire study population, the mean baseline serum uric acid was 5.5 mg/dL, and this value did not change significantly on the second visit (Table 2). Patients in the pre-specified “large reduction” group had an absolute mean serum uric acid decrease of 0.21 mg/dL on the second visit, with a mean percent decrease of 2.7%; patients in the pre-specified “large increase” group had an absolute mean serum uric acid increase of 0.19 mg/dL, with a mean percent increase of 4.6%; patients in the pre-specified “moderate reduction” group had an absolute mean serum uric acid decrease of 0.04 mg/dL on the second visit, with a mean percent decrease of 0.24%; and patients in the pre-specified “moderate increase” group had an absolute serum uric acid mean increase of 0.12 mg/dL, with a mean percent increase of 3% (Figure 1). 

**Figure 2:**
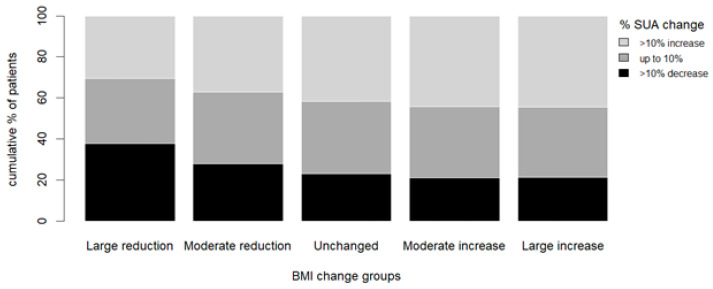
Serum uric acid change percentage according to the pre-specified BMI change groups. Bars represent the pre-specified BMI change groups; different shadings represent the percent of patients in each prespecified group with at least a 10% increase in UA levels from visit 1 to visit 2 (light grey), up to 10% change in UA levels from visit 1 to visit 2 (dark grey), and 10% decrease in UA levels from visit 1 to visit 2 (black). Abbreviations: BMI, body mass index; SUA, serum uric acid.

The proportion of patients with at least a 10% increase in serum uric acid increased with the relative change in BMI (17.3%, 20.8%, 23.4%, 27.5%, and 33.1% for “large reduction”, “moderate reduction”, “unchanged”, “moderate increase”, and “large increase” groups, respectively (*p* < 0.01)). Comparably, the proportion of patients with at least a 10% decrease in serum uric acid decreased with the relative change in BMI (31.2%, 22%, 17.6%, 15.2%, and 14.2% for “large reduction”, “moderate reduction”, “unchanged”, “moderate increase”, and “large increase”, respectively (*p* < 0.01)) (Figure 2).

**Figure 3:**
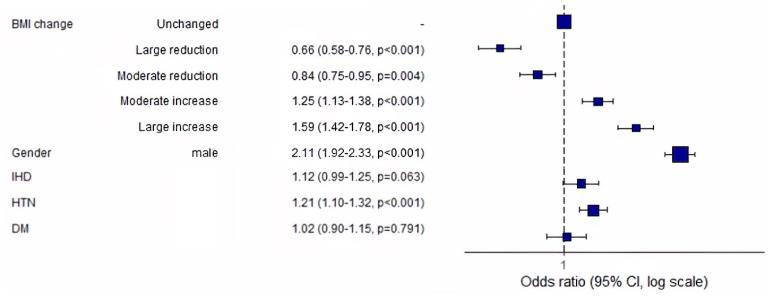
Association between patients’ baseline characteristics and increases of at least 10% in serum uric acid levels on visit 2. Abbreviations: BMI, body mass index; IHD, ischemic heart disease; HTN, hypertension; DM, diabetes mellitus.

Compared to the “unchanged” group, the odds ratios for serum uric acid increases of at least 10% were 0.66, 0.84, 1.25, and 1.59 for the “large reduction” (*p* < 0.001), “moderate reduction” (*p* = 0.014), “moderate increase” (*p* < 0.001), and “large increase” (*p* < 0.001) groups (Figure 3). Male gender and hypertension were significantly associated with an increased odds ratio for an at least 10% increase in serum uric acid levels (Figure 3). Subgroup analyses by gender, baseline BMI, and hypertension showed similar results. 

## 4. Discussion

We have demonstrated a significant association between non-interventional weight changes and alterations in serum uric acid levels. Serum uric acid was progressively lower as BMI decreased and progressively higher as it increased. The proportion of patients whose serum uric acid levels were increased by at least 10% between visits increased with the relative increase in BMI and the proportion of patients whose serum uric acid levels were decreased by at least 10% decreased with the relative decrease in BMI.

The association between obesity and serum uric acid is well established. Obesity highly predisposes for hyperuricemia, partially due to the overproduction of uric acid in adipose tissue, as well as decreased urinary uric acid clearance [36], while lower body weight is associated with a lower prevalence of hyperuricemia [37]. Some suggest that the association between BMI and serum uric acid is mediated by hyperinsulinemia or insulin resistance [38]. Most studies, to date, have focused on the association between baseline BMI and serum uric acid, or on the effects of interventional weight loss regimens on serum uric acid among overweight and obese patients [6,11,37,39,40]. Notably, a significant correlation was recently observed between decreases in fat mass and lower serum uric acid among normal-weight Korean individuals [41].

Changes in BMI following targeted weight management interventions have previously been shown to affect both cardiovascular outcomes and overall mortality [3,41,42,43]. Specific dietary interventions, not directly aimed at lowering serum uric acid levels, have also been shown to be beneficial in both lowering serum uric acid, as well as reducing cardiovascular risk in a weight-dependent manner [30]. A recent study has demonstrated that a combination of a high triglycerides-glucose index and high serum uric acid levels have a synergic effect on mortality risk [44]. Interestingly, we have recently shown that the triglycerides-to-high-density-lipoprotein-cholesterol ratio (TG/HDL-C) is closely associated with minor non-interventional weight changes. Therefore, these data, along with our current findings, further highlight the importance of minor weight changes, as well as triglycerides and serum uric acid, in cardiovascular risk stratification [33,34]. In a multivariable regression analysis, we found that male sex and hypertension, which are traditional cardiovascular risk factors, were also associated with an increase in serum uric acid, further emphasizing the association of serum uric acid with classic metabolic risk factors, as well minor non-interventional weight changes. 

Our study has several limitations. First, this is a retrospective study. Therefore, causality between BMI alterations and serum uric acid could not be established. However, our large cohort has the potential to mitigate the impact of this limitation. In addition, we lack individual dietary data for our cohort, which could potentially be a source for the confounding effects of different diets on weight change and cardiovascular health. Nevertheless, the influence of various diet compositions on weight loss is somewhat controversial. Studies have shown that reduced-calorie diets result in clinically meaningful weight loss regardless of which macronutrients are consumed [45] and that the difference found between various carbohydrate–fat content combinations was not sustained three months after nutritional intervention [46]. Thus, we believe that the specific diet composition does not have a confounding impact on our findings. 

## 5. Conclusions

In conclusion, we show that non-interventional weight alterations, even when modest, are associated with a significant change in serum uric acid levels. Since serum uric acid is an emerging cardiovascular risk factor, our findings, together with data from interventional weight programs, further highlight the importance of weight changes on serum uric acid and possibly on cardiovascular risk stratification and the prevention of cardiovascular-related morbidity and mortality. 

## Figures and Tables

**Table 1:** Baseline characteristics.

	Large Reduction	Moderate Reduction	Unchanged	Moderate Increase	Large Increase	Total	*p* Value
	(*n* = 1791)	(*n* = 2208)	(*n* = 10,713)	(*n* = 2718)	(*n* = 1763)	(*n* = 19,193)	
Gender							<0.001
Male	1198 (67%)	1625 (74%)	7998 (75%)	1987 (73%)	1101 (62%)	13,909 (72%)	
Female	593 (33%)	583 (26%)	2715 (25%)	731 (27%)	662 (38%)	5284 (28%)	
Age (years)	49 (±10)	50 (±11)	50 (±10)	49 (±10)	48 (±9.9)	50 (±10)	<0.001
IHD	205 (11%)	269 (12%)	1280 (12%)	289 (11%)	171 (10%)	2214 (12%)	0.0301
HTN	515 (29%)	679 (31%)	3115 (29%)	738 (27%)	498 (28%)	5545 (29%)	0.0821
DM	191 (11%)	226 (10%)	1018 (10%)	263 (10%)	156 (9%)	1854 (10%)	0.337

Abbreviations: IHD, ischemic heart disease; HTN, hypertension; DM, diabetes mellitus.

**Table 2:** Body mass index and uric acid levels.

	Large Reduction	Moderate Reduction	Unchanged	Moderate Increase	Large Increase	Total	*p* Value
	(*n* = 1791)	(*n* = 2208)	(*n* = 10,713)	(*n* = 2718)	(*n* = 1763)	(*n* = 19,193)	
First visit BMI (kg/m^2^)	28 (±4.5)	26 (±3.7)	26 (±3.7)	26 (±3.8)	25 (±4.0)	26 (±3.8)	<0.001
Second visit BMI (kg/m^2^)	25 (±3.8)	25 (±3.5)	26 (±3.7)	26 (±3.9)	27 (±4.5)	26 (±3.8)	<0.001
Absolute BMI change (kg/m^2^)	−2.5 (±1.7)	−0.95 (±0.24)	0.013 (±0.33)	0.91 (±0.23)	2.0 (±1.5)	−0.014 (±1.3)	<0.001
%BMI change	−8.6 (±4.2)	−3.6 (±0.70)	0.059 (±1.3)	3.6 (±0.71)	8.1 (±6.2)	0.070 (±4.7)	<0.001
First visit SUA (mg/dL)	5.5 (±1.4)	5.5 (±1.3)	5.5 (±1.3)	5.4 (±1.4)	5.3 (±1.4)	5.5 (±1.4)	<0.001
Second visit SUA (mg/dL)	5.3 (±1.4)	5.5 (±1.3)	5.5 (±1.4)	5.6 (±1.4)	5.4 (±1.4)	5.5 (±1.4)	<0.001

Abbreviations: BMI, body mass index; SUA, serum uric acid.

## Data Availability

The datasets generated during and/or analyzed during the current study are available from the corresponding author on reasonable request.
